# Community Structure of Macrobiota and Environmental Parameters in Shallow Water Hydrothermal Vents off Kueishan Island, Taiwan

**DOI:** 10.1371/journal.pone.0148675

**Published:** 2016-02-05

**Authors:** Benny Kwok Kan Chan, Teng-Wei Wang, Pin-Chen Chen, Chia-Wei Lin, Tin-Yam Chan, Ling Ming Tsang

**Affiliations:** 1 Biodiversity Research Center, Academia Sinica, Taipei, Taiwan; 2 Department of Life Science, Tunghai University, Taichung, Taiwan; 3 Department of Exhibition, National Museum of Marine Biology and Aquarium, Pingtung, 944, Taiwan; 4 Institute of Marine Biology, National Taiwan Ocean University, Keelung, Taiwan; 5 Center of Excellence for the Oceans, National Taiwan Ocean University, Keelung, Taiwan; National Taiwan Ocean University, TAIWAN

## Abstract

Hydrothermal vents represent a unique habitat in the marine ecosystem characterized with high water temperature and toxic acidic chemistry. Vents are distributed at depths ranging from a few meters to several thousand meters. The biological communities of shallow-water vents have, however, been insufficiently studied in most biogeographic areas. We attempted to characterize the macrofauna and macroflora community inhabiting the shallow-water vents off Kueishan Island, Taiwan, to identify the main abiotic factors shaping the community structure and the species distribution. We determined that positively buoyant vent fluid exhibits a more pronounced negative impact to species on the surface water than on the bottom layer. Species richness increased with horizontal distance from the vent, and continuing for a distance of 2000 m, indicating that the vent fluid may exert a negative impact over several kilometers. The community structure off Kueishan Island displayed numerous transitions along the horizontal gradient, which were broadly congruent with changes in environmental conditions. Combination of variation in Ca^2+^, Cl^-^, temperature, pH and depth were revealed to show the strongest correlation with the change in benthic community structure, suggesting multiple factors of vent fluid were influencing the associated fauna. Only the vent crabs of Kueishan Island may have an obligated relationship with vents and inhabit the vent mouths because other fauna found nearby are opportunistic taxa that are more tolerant to acidic and toxic environments.

## Introduction

Hydrothermal vents are located along submarine ridges and are active geothermal areas with hot fluid emitting from the vent mouths [1, 2]. The emitted fluid typically contains a large amount of sulfur compounds and carbon dioxide formed from heat driven chemical reactions and metals leached from rocks [1]. The surrounding water chemistry is strongly influenced by these conditions, leading to an acidic environment. Hydrothermal vents are distributed at depths ranging from a few meters to more than 5000 m throughout the world’s oceans [1–3]. Deep-sea hydrothermal vents receive no solar radiation and rely solely on chemosynthesis for the energy supply. Accordingly, deep-sea hydrothermal vents are sometimes considered to reflect the life forms and chemosynthetic ecosystem of an early planet [4, 5]. Thus, the ecology of deep-sea hydrothermal vents has been actively studied recently [1, 2].

Shallow-water vent research has a longer history than deep-sea vent research, dating back to the middle 1880s [3, 6]. Investigations on the ecology of shallow-water hydrothermal vents have been conducted in many regions, including Papua New Guinea, Greece, the Kurlie Islands, Italy, Baja California, Japan, and Taiwan [3, 6, 7]. Data have suggested that shallow and deep-sea vents exhibit major differences in their physical and chemical properties, thereby resulting in dissimilar associated biological communities. Most notably, shallow-water hydrothermal vents contain substantially fewer fauna which have an obligated relationship with the vents than their deep-water counterparts. Furthermore, since shallow-water vents occur in the euphotic zone, the contribution of photosynthesis to primary production is important, whereas at deep-sea vents most energy is believed to be generated by chemosynthesis [3, 6, 7]. However, studies on shallow-water vent ecology remain few, leaving their biodiversity mostly unexplored, hampering the understanding of ecosystem functioning in these habitats.

**Fig 1:**
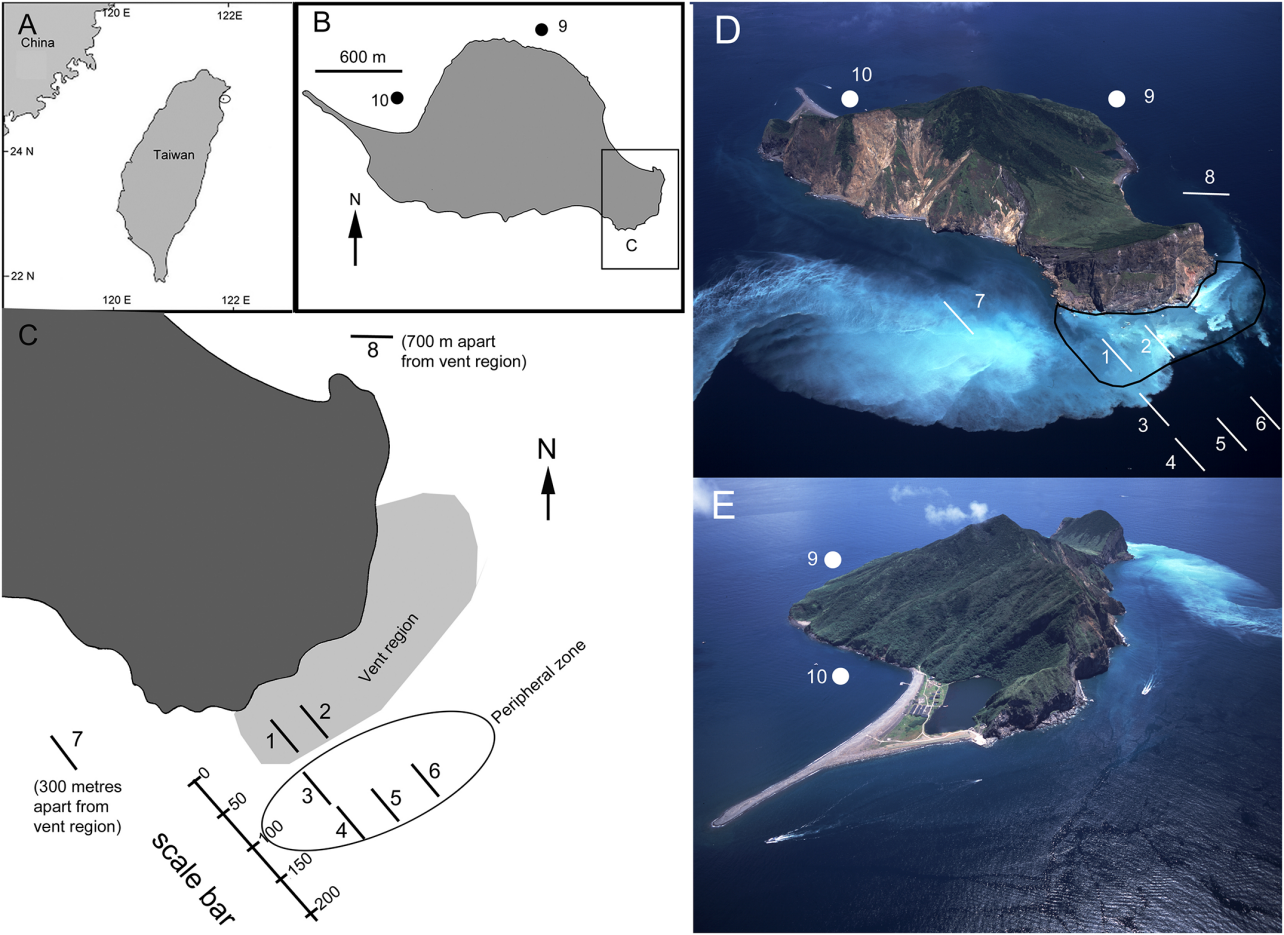
(A) Map of Taiwan showing the location of Kueishan Island in the northeastern coast. (B) Map of Kueishan Island showing the location of Stations 9 and 10. (C) Southeast water off Kueishan Island showing the vent region, peripheral region and the stations of the present study. Stations 1–8 are 50 m transect extending to the SE. (D) Aerial photo of Kueishan Island, showing the location of vent region and the stations. (E) Aerial photo of northwest coast of Kueishan Island. Reprinted from Northeast and Yilan Coast National Scenic Area Administration under a CC BY license, with permission from Ministry of Transportation and Communication, Taiwan, R.O.C., original copyright [2015]. Scales in metres.

Kueishan Island is located approximately 11 km from the northeastern coast of Taiwan (Fig 1A). Kueishan Island is renowned for having numerous gaseohydrothermal vents (10–300 m deep) and the most acidic vents (with lowest recorded water pH at the vent) in the world (Fig 1) [8, 9]. Gases produced by the vents are mainly composed of carbon dioxide (92%) and a small amount of hydrogen sulfide [10]. The fluids from the vent float to the sea surface and circulate with tidal currents (Fig 1D and 1E) [9]. The substratum of the vent stations are composed of pure sulfur sands and native sulfur balls, formed by the reaction of hydrogen sulfide and sulfur dioxide [11]. Metagenomic characterization of the bacterial community at the vent smokers and the surface water has revealed a high abundance of chemosynthetic bacteria [10]. Studies regarding macrobiota diversity in these shallow-water vents are limited. Chen et al. briefly reported the species inhabiting the shallow-water hydrothermal vents at Kueishan Island, including the vent crab *Xenograpsus testudinatus*, “a snail”, “a sea anemone”, a “sipuncula”, and one species of “fish” [8, 9]. Recently, Chen et al. [12] reported that there are abundant populations of the snail *Anachis misera* (Sowerby, 1844) close to the vent regions in the Kueishan Island. However, the species identities of most inhabitant remain un-recognized and there is no report on the status of primary producers in Kueishan Island.

Shallow-water vent ecosystems are based on energy supplied by both photosynthesis and chemosynthesis and exhibit toxic effects in the surrounding water. Hence, a detailed understanding of the diversity gradient around the vents would show how the vent proximity affect benthic biota diversity off Kueishan Island. The present study characterizes the macrofauna and macroflora of the community inhabiting the shallow-water vents off Kueishan Island. We also examined the changes in physical and chemical water properties along an environmental gradient extending horizontally from the vent smokers. The major objective was to determine the composition and abundance of different species near the vents to provide more insights into the trophic structure, functional groups, and interaction of the organisms in shallow-water vent systems. Furthermore, we aimed to identify the main abiotic factors shaping the community structure and the species distribution in the shallow-water vent area.

## Materials and Methods

### Study sites and timing

**Fig 2:**
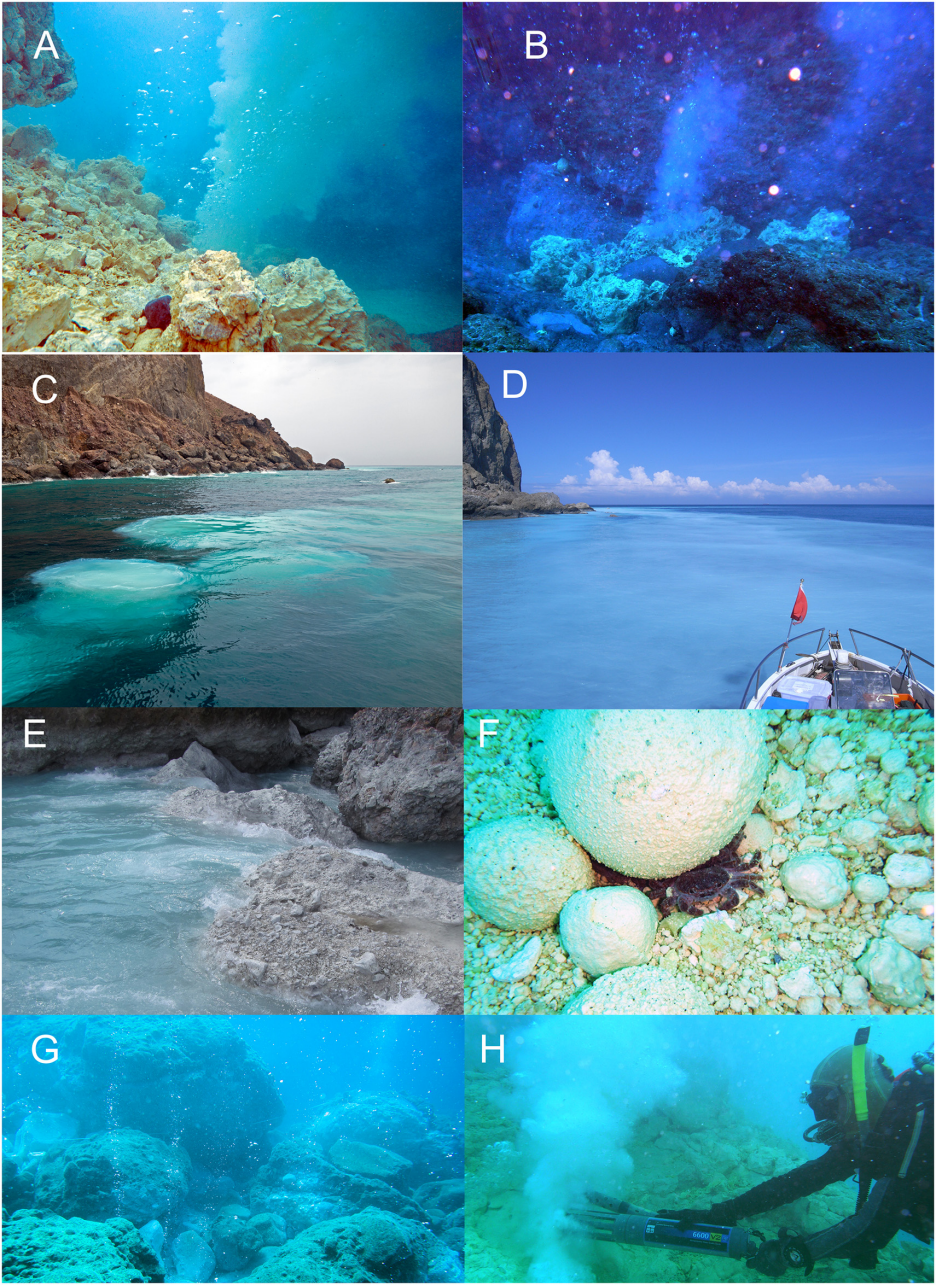
Photos of environments in Kueishan Island. (A) Underwater photo of a large yellow vent with jet of fluid discharge. The yellow vents were surrounded by numerous sulfur blocks and moulds. (B) Underwater photo of white vent which have smaller volume of fluid discharge compared to yellow vent. (C) The white circular patches of vent fluid on the water surface above vent (Station 1). (D) The fluid float up from the yellow and white vents are mixed and dispersed by currents, resulting in white colour in a large area of surface water off Kueishan. (E) The rocky intertidal close to the vent region was coated by a thick white film. (F) Substratum of the vent region contained a lot of sulfur balls and sands, with vent crabs *Xenograpsus testudinatus* often hiding underneath. (G) The occasional sparse bubbling of vent gas from bottom of the sea in the peripheral region (Station 5). (H) *In-situ* measurement of physicochemical parameters of the vents using autonomous YSI probes.

Samplings of the species were collected by 18 SCUBA dives from June to July 2014 and two SCUBA dives in May 2015 (Table 1). No specific permission is required for field survey in Kueishan Island and the study did not involve endanger or protected species. Samplings of the water chemistries of all stations were conducted from July 28–29, 2014. In the southeastern waters off the island, there is a vent area containing >30 examples of two major types of hydrothermal vent distributed at 10–30 m (Figs 1, 2A and 2B). The two types of vent differ in the temperature and acidity of the fluids, as well as the discharge flow rate [8]. High temperature vents (generally referred to as yellow vents; Fig 2A) often form pure sulfur mounds or chimneys, producing strong jets of elemental sulfide yellowish water. At the station, the flow rate of these vents was reported can up to 158 t h^-1^ [8, 9]. The water temperature of the fluids can reach 92–116°C with pH values of 1.5–6.3 [8, 9]. Low temperature vents (generally referred to as white vents; Fig 2B) are frequently observed in rock crevices at the station and have fluid temperatures of 48–62°C with pH levels of 1.8–7.0, producing weaker jets of fluid (flow rate = 7 t h^-1^) [8, 9]. This major vent region is approximately 75,000 m^2^ in area (Fig 1D and 1E).

**Table 1:** Locations, depth of stations and dates of the transects for biological assemblage investigation in the present study. The longitude and latitude were recorded at the start of each transect.

Station	Region	Longitude, Latitude	Mean. depth (m)	Dates of survey
1	Vent region	24.50.112N, 121.57.741E	10	5 June and 14 July, 2014
2	Vent region	24.50.087N, 121. 57.709E	10	14 July, 2014
3	Peripheral region	24.50.054N, 121.57.723E	13	14 July, 2014
4	Peripheral region	24.50.027N, 121.57.725E	13	28 July, 2014
5	Peripheral region	24.50.056N, 121.57.752E	15	28 July, 2014
6	Peripheral region	24.50.058N, 121.57.779E	14	28 July, 2014
7	300m from vent region	24.50.088N, 121.57.576E	15	29 July, 2014
8	700 m from vent region	24.50.495N, 121.57.850E	18	29 July, 2014
9	1500 m from vent region	24.50.838N, 121.57.574E	11	17 April 2015
10	>2000 m from vent region	24.50.781N, 121.56.680E	10	17 April 2015

In the present study, a total of 10 stations (depths ranging from 10–20 m) were investigated in the waters surrounding Kueishan Island (Fig 1A–1D; Table 1). Average depth of each station was calculated from the depth data recorded in every 10 minute interval by an autonomous YSI 6600V2 probe during SCUBA diving in the stations. Stations 1 and 2 (average depth 10 m) were located within the major vent region (Fig 1). One large yellow vent (chimney = 1 m tall, diameter >40 cm) and several white vents were at Station 1, and several white and yellow vents were at Station 2 (Fig 2D). The substratum of Stations 1 and 2 were characterized by sulfur sands, with large-sized irregularly shaped sulfur balls [11] on the surface of the sand (Fig 2F). Stations 3–6 (average depth ranged from 12–14 m, see Table 1) were 150–300 m off the southeast coastline of Kueishan Island and located in the peripheral area of the major vent region (Fig 1). Large vents were not discovered and only occasional fine bubbling from the substratum was observed at the stations within the peripheral zone (Fig 2G).

The remaining four stations (Stations 7–10) were located a minimum of 300 m from the vents (Station 1). Station 7 was located 300 m southwest of Station 1, and Station 8 was located approximately 700 m Station 1 (Fig 1C); these two stations were considered as the rim of the peripheral region. Stations 9 and 10 were located 1500 m and >2000 m, respectively, westward from the vent region. A high coverage of coral reefs was observed at Stations 9 and 10, suggesting that the vent discharge impact was minimal-none (the flow of the vent effluents are directed towards E or NE direction due to the strong Kuroshio currents; Fig 1D)[13]; these two stations served as non-vent, control stations. Underwater transect samplings were conducted for all 10 stations. A 50-m transect running in a southeast direction from the vent was established for Stations 1 and 2. For Stations 3–8, the starting point of the transect was selected to be at 10 m and then running in a southeast direction. In Stations 9 and 10, the transects were established from 10–15 m, horizontal to the coastline of the island. Water, macrofauna, and macroflora sampling being conducted along the transect of each station (Fig 1; Table 1).

### Physical and chemical parameters of the vent region

We conducted hierarchical analyses of the water chemistries, comparing that of the vent mouth to that of the various stations. First, we based on Station 1 (vent station) to determine any variation in water chemistries between the fluid discharged from the vent mouth, water surrounding the vent mouth (approximately 1 m horizontally from the vent mouth), and surface water at the top of the vent. Subsequently, we analyzed the vertical differences of the 10 stations by comparing the water properties of the surface and bottom water layers. Finally, the water chemistries among stations were compared to evaluate any physical and chemical gradients across the stations.

### Physicochemical parameters

To examine the spatial variations of physico-chemical parameters within the vent Station 1 (vent mouth, 1 m apart from vent mouths and surface water above vents), 2-L water samples were collected from the mouths of the three yellow and three white vents, and three water samples were collected at 1 m from each of these vents. A further three surface water samples were also collected where the fluids from both type of vents was mixed; therefore, only three 2-L water samples were collected at the surface above the vent region (Σn for all water samples in Station 1 = 3 x yellow vent mouths + 3 x 1 m apart from yellow vent mouth + 3 x white vent mouths + 3 x 1 m apart from white vent mouth + 3 x surface water = 15).

To examine the spatial variation in physico-chemical parameters among stations, we measured the water temperature, pH, dissolved oxygen, and salinity levels of the surface and bottom water layers of each station (at the start point of the transect) in situ, using an autonomous YSI 6600V2 probe while SCUBA diving (Fig 2H). Triplicate measurements were taken approximately 1 m apart in both the surface and bottom water layers. The variations in parameters between the surface and bottom layers and between stations were tested using a two-way analysis of variance (ANOVA). Dataset passed the equal variance tests (Sigma Stat version 3.5, SPSS). Finally, 2-L water samples were collected *in situ* at the 0, 25, and 50-m points of the transects at Stations 1–10 for analysis of physico-chemical parameters.

All water samples were stored in gas tight bottle and kept in icebox filled by ice during transportation (<3 hours of collection). pH, S^2-^, SO_4_^2-^ and NO_3_^-^ were analyzed immediately upon arrival at the laboratory. The pH of the water samples were examined using a desktop pH meter (Suntax SP-2100)_ (±0.01) at room temperature. Concentrations of common components of vent fluid, including sulfur compounds, cations, and anions (Al^3+^, Ca^2+^, Cl^-^, Fe^2+^, Fe^3+^, Mg^2+^, Mn^2+^, NO_3_^-^, PO_4_^2-^, S^2-^, SiO_2_, and SO_4_^2-^) [4], were analyzed using a Merck Pharo 2000 spectrophotometer and kit according to standard Merck Spectroquant protocols (Details of the product number and sensitivity, see S1 Table) [14]. Arsenic was measured from single water samples collected from each stations, using Hydride Generation Atomic Absorption Spectrometry (American Public Health Association, American Water Works Association and Water Pollution Control Federation, 2012), conducted by Precia Environment Corporation, Ltd Taiwan. Variations in pH, ion concentrations in different water samples were analysed using one-way analysis of variance (ANOVA) for each physicochemical parameter, and significant factor was further analysed using SNK tests.

### Biological assemblage survey

At Stations 1–10, three 25 × 25 cm quadrats were placed haphazardly every 5 m along the 50 m transect, and the quadrats were photographed (Σn = 33). Counts were made of the number of mobile individuals inside the quadrat based on the photograph although these may be underestimated because some moved when the quadrats were placed). The percentage of coverage of sessile species was scored using Sigma Scan Pro image software (version 5.0, SPSS). The average abundance of each species was calculated from the 33 quadrats of each station. Identifications of crustaceans, molluscs, algae and other invertebrates were based on Segawa [15], Miyake [16, 17], Okutani [18] and Dai and Horng [19, 20].

### Statistical Analyses

The variation in water chemical parameters along the vent mouth, a 1-m region from the vent mouth, and the surface water were analyzed using a one-way analysis of similarities (ANOSIM). In PRIMER Version 6 (Plymouth Routines in Multivariate Analysis; PRIMER-E, Ltd.) and the environmental parameters were normalized using the normalize routine in PRIMER 6, and then a similarity matrix for the seawater parameters based on a comparison between the examined locations was assessed using a Euclidean distance similarity test followed by nonmetric multidimensional scaling (nMDS) plots [21].

The variation in chemical and physical parameters and biological assemblages among the 10 stations was analyzed using ANOSIM. The density or percentage coverage data of was square root transformed, and a similarity matrix based on the Bray—Curtis similarity index [22] was conducted on each sample pair and a nMDS ordination plot was generated to visualize the ordination distribution [23]. An ANOSIM was also conducted to examine the variations in species community among the sampling regions: the vent region (Stations 1 and 2), peripheral region (Stations 3–6), 300–700 m from the vent region (Stations 7 and 8) and control stations (Stations 9 and 10, 1500–2000 m from vent region). Significance was evaluated based on the generated global *R* and *p* values.

Finally, the BVSTEP procedure was conducted on the environmental variables to select a subset of the environmental variables with a multivariate sample pattern that matched the complete set of environmental variables. This subset was then used for the BIO-ENV analysis [24]. Following this, the relationships between the species assemblage and the chemical parameters were estimated using BIO-ENV routines [25] in PRIMER, which to determines the best combination of environmental parameters for correlating the observed pattern of biological data.

## Results

### Physicochemical environment of the vent region

The fluid produced from the vents floats on the water surface, forming circular white colored patches (Fig 2C). The white fluid is dispersed by the surface current, resulting in the whitish cloudy appearance of the surface water and rocks off the southeast coast of Kueishan Island (Fig 2D and 2E). In Station 1 (the vent region), pH of the fluids collected from the mouths of yellow and white vents, waters from 1 m apart from yellow and white vents and from surface water was significantly different (F (4, 10) = 152.5, *p* < 0.05). Post-hoc SNK tests indicated pH value of the hydrothermal vent fluid from the yellow vents (mean ± 1SD = 4.5± 1.0) was significantly lower than that of the white vents (mean = 5.9 ± 0.3) (S1 Fig). The water at 1 m adjacent to both the yellow and white vents showed a significant higher pH value (mean = 7.3 ± 0.13 and 7.3 ± 0.18 respectively) than the fluids from the mouths of yellow and white vents. The pH value of the surface water above the vent region (6.2 ± 0.03) was significantly lower than the waters at 1 m apart from the vents (1 m from yellow vents: 7.32 ± 0.13, 1 m from white vents: 7.32 ± 0.18) (S1 Fig). pH value of surface water above vent region is significantly lower from the mouth of yellow vents but not at the white vent (S1 Fig). Furthermore, the ion concentration also exhibited significantly difference among the fluid from yellow vents, white vents, the water 1 m apart from the vent mouth and the water at the surface of the vent (see more details in S1 Text).

**Fig 3:**
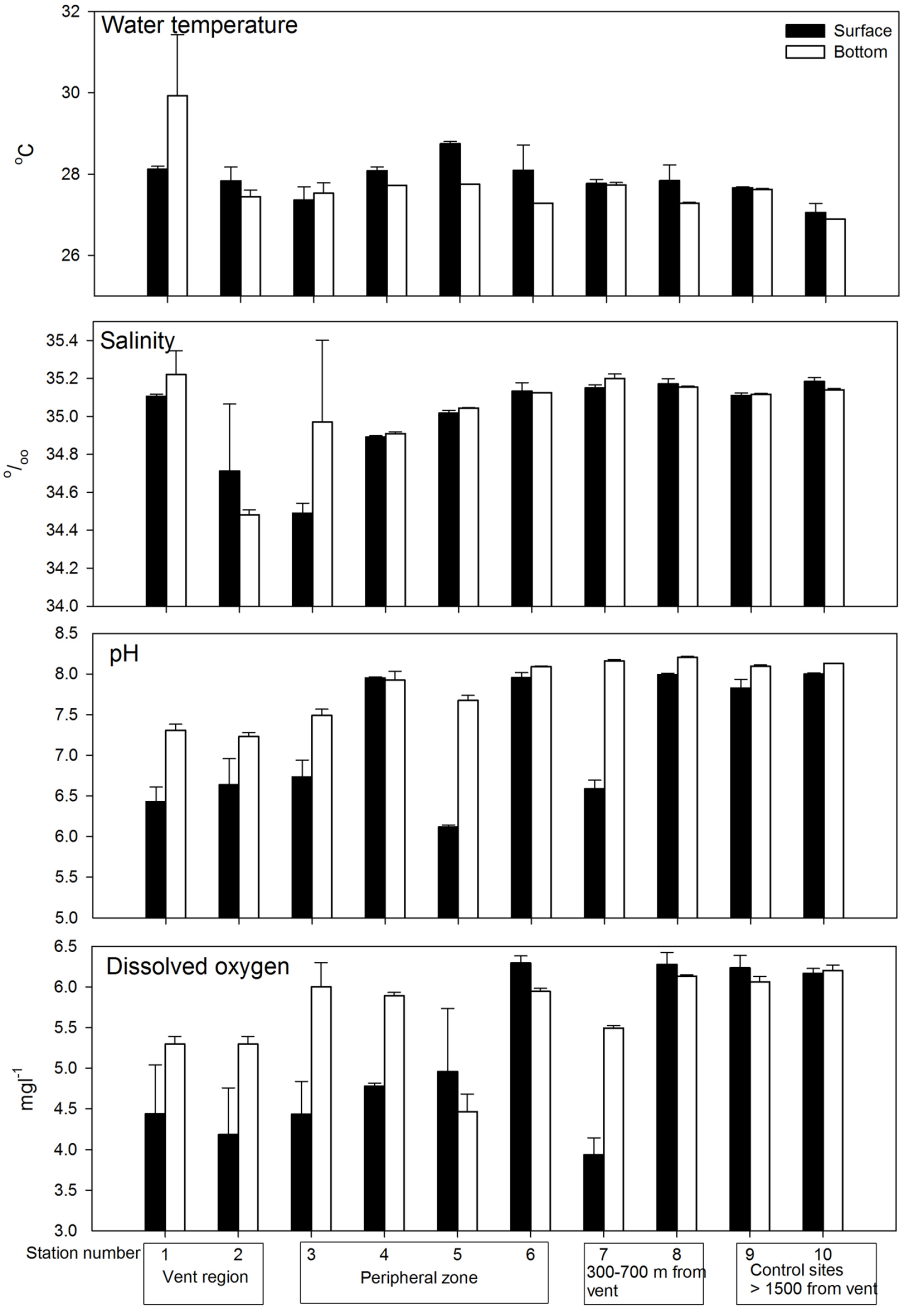
Vertical differences (surface and bottom) of mean (±1SD) water temperature, salinity, pH value and dissolved oxygen in all 10 stations.

Results from two-way ANOVA indicated water temperature, salinity, pH and dissolved oxygen have significant differences in the interactions between stations and depths (Table 2). SNK pairwise comparisons suggested Station 1 showed a significantly higher bottom temperature (average 30.0 ± 1.5°C) than the surface water (28 ± 0.01°C) (Fig 3; Table 2). By contrast, the surface water exhibited a significant higher temperature than the bottom at Stations 4 (surface 28.0 ± 0.09°C, bottom 27.7 ± 0.001°C) and 5 (surface 28.7 ± 0.05°C, bottom 27.7 ± 0.01°C). The surface and bottom water temperature were not significantly different at the other stations (Fig 3). Salinity did not show vertical differences at most of the stations in the SNK tests, with the exceptions of a higher salinity in the bottom water layer at Station 3 (surface 34.5 ± 0.05, bottom 35.0 ± 0.04) and vice versa at Station 2 (surface 34.7 ± 0.3, bottom 34.5 ± 0.02) (Fig 3; Table 2). The pH values displayed strong vertical differences at most stations within or close to the major vent area (Stations 1, 2, 4, and 5), with the surface waters being significantly having lower pH (6–6.8) than the bottom water (7.4–7.8) (Fig 3; Table 2). By contrast, the pH values of the surface and bottom waters were similar in locations with increasing horizontal distance from the major vent region (Stations 6–10) (Fig 3; Table 2). Dissolved oxygen levels exhibited significant vertical differences at stations 1, 2, 3, 4, and 7, with higher dissolved oxygen levels at the surface layers (5.3–6.0 mgl^-1^) than the bottom layers (3.9–4.9 mgl^-1^). There were no vertical differences in dissolved oxygen concentrations at the control stations (Stations 8–10) (Fig 3; Table 2).

**Table 2:** Two way ANOVA and SNK tests to investigate the variations in water temperature, salinity, pH value and dissolved oxygen between surface and bottom (factor Vertical differences), and among the 10 stations extending from the vent region in Kueishan Island.

	*Df*	MS	*F*	P value
*Water temperature*				
Station	9	2.4	22.9	0.001*
Vertical differences	1	0.06	0.6	0.4
Station x Vertical differences	9	1.4	13.5	0.001*
Residual	40	0.1		
SNK tests				
Stations:	S> B: 4, 5 S = B: 2, 3, 6, 7, 8, 9, 10 B > S: 1,			
Surface:	4 = 5>1 = 2 = 3 = 6 = 7 = 8 = 9>10			
Bottom:	1 > 2 = 3 = 4 = 5 = 6 = 7 = 8 = 9 = 10			
*Salinity*				
Station	9	4.54	30.08	0.001*
Vertical differences	1	0.25	14.93	0.001*
Station x Vertical differences	9	4.23	27.96	0.001*
Residual	40	0.67		
SNK tests				
Stations:	S> B: 2; S = B: 1, 4, 5, 6, 7, 8, 9, 10; B > S: 3,			
Surface:	10 = 7 = 6 = 5>9 = 8 = 4 = 3 = 2 = 1			
Bottom:	1>7 = 8>2 = 3 = 4 = 5 = 6 = 9 = 10			
*pH*				
Station	9	2.56	216	0.001*
Vertical differences	1	0.3	26	0.001*
Station x Vertical differences	9	0.33	27.8	0.001*
Residual	40	0.0119		
SNK tests				
Stations:	S > B: 3; S = B: 6, 7, 8, 9, 10; S < B: 1, 2, 4, 5,			
Surface:	6 = 8 = 9 = 10>1 = 2 = 3 = 4 = 5 = 7			
Bottom:	4 = 6 = 7 = 8 = 9 = 10>1 = 2 = 3 = 5			
*Dissolved oxygen*				
Station	9	24.28	31.07	0.001*
Vertical differences	1	4.67	53.78	0.001*
Station x Vertical differences	9	10.12	12.96	0.001*
Residual	40	3.43		
SNK tests				
Stations:	S > B: 6; S = B: 8, 9, 10; S < B: 1, 2, 3, 4, 7			
Surface:	6 = 8 = 9 = 10>4 = 5>1 = 2 = 3>7			
Bottom:	6 = 8 = 9 = 10>1 = 2 = 3 = 4>5 = 7			

* = significant.

**Fig 4:**
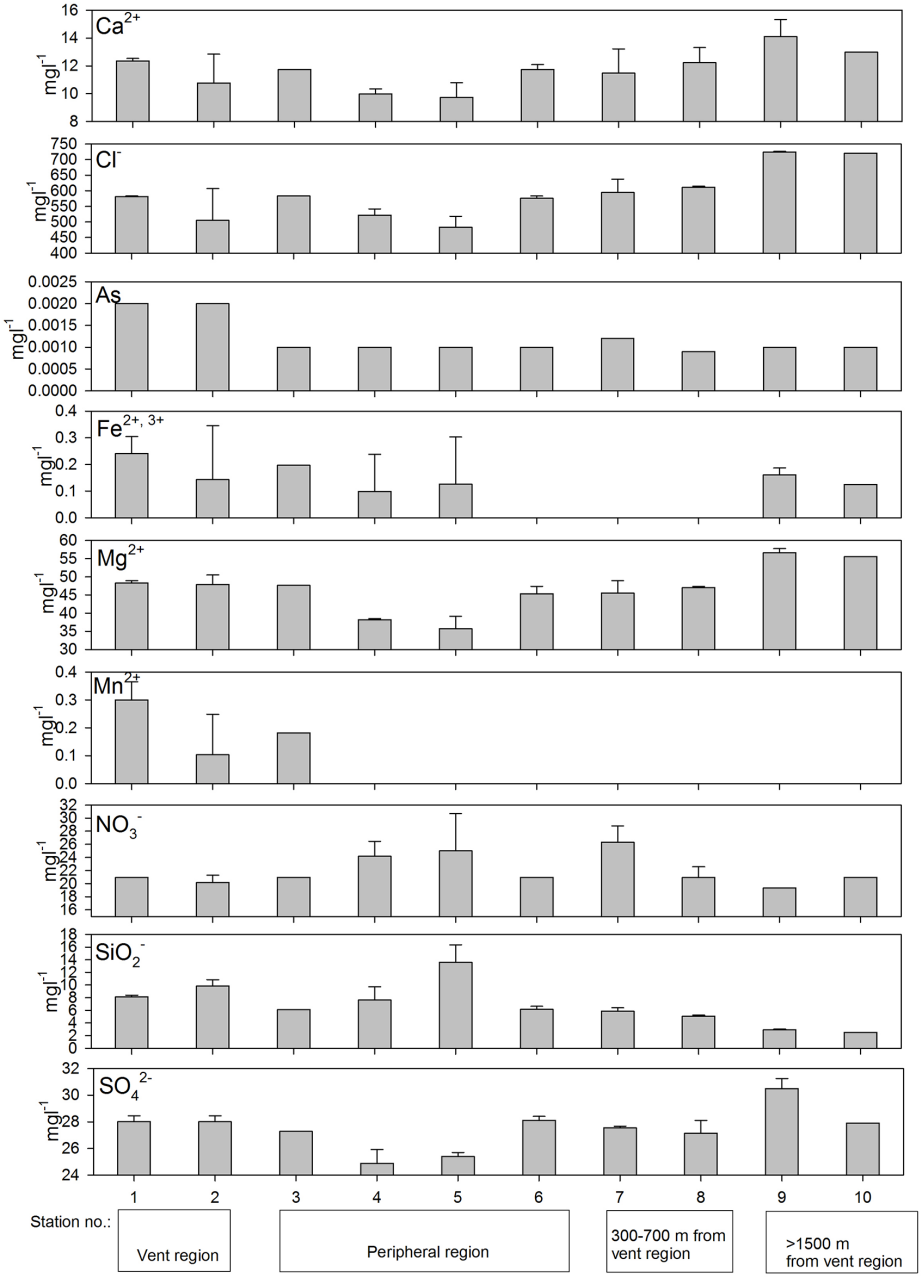
Variation in chemical parameter (mean ±1SD) along the spatial scales from Stations 1–10.

Along the spatial scale extending from the major vent area, the water temperature was homogenous across waters off Kueishan Island (Fig 3). There was no spatial gradient in salinity along the stations departing from the vent region. However, the stations closer to the vent area generally exhibited lower pH values (stations 1–2: bottom = 7.3–7.23; surface = 6.42–6.63) and dissolved oxygen concentrations (stations 1–2: bottom = 4.2–4.4 mgl^-1^; surface = 4.18–4.43) compared with those of the stations at the stations at 300–700 apart from the vents and the control stations (stations 7–10: pH at bottom = 8.1–8.2 and surface = 6.58–7.99, dissolved oxygen at bottom = 5.4–6.2 mgl^-1^ and surface = 3.93–6.16) (Fig 3; Table 2). By contrast, the concentrations of sulfur and other ions measured displayed high variations among stations, and no clear trend could be inferred for most of them (Fig 4). Mn^2+^ was the only cation that was only present in the stations 1–3 but absent from the other stations. Fe^2+^ and Fe^3+^ occurred in the vent and nearby regions (Stations 1–5), as well as at the non-vent stations (Stations 9 and 10), but could not be detected from the region in between (Stations 6–8) (Fig 4). Noticeably, the concentrations of arsenic were higher in Station 1 and 2 (vent stations) than the other non-vent stations. Yet only one water sample from each stations was analyzed for the arsenic that the reliability of this observation need further verification. The nMDS plot of the water chemistries revealed that Stations 1, 2–3, 4–8 and 9–10 form four distinct clusters.

### Biological assemblages

**Fig 5:**
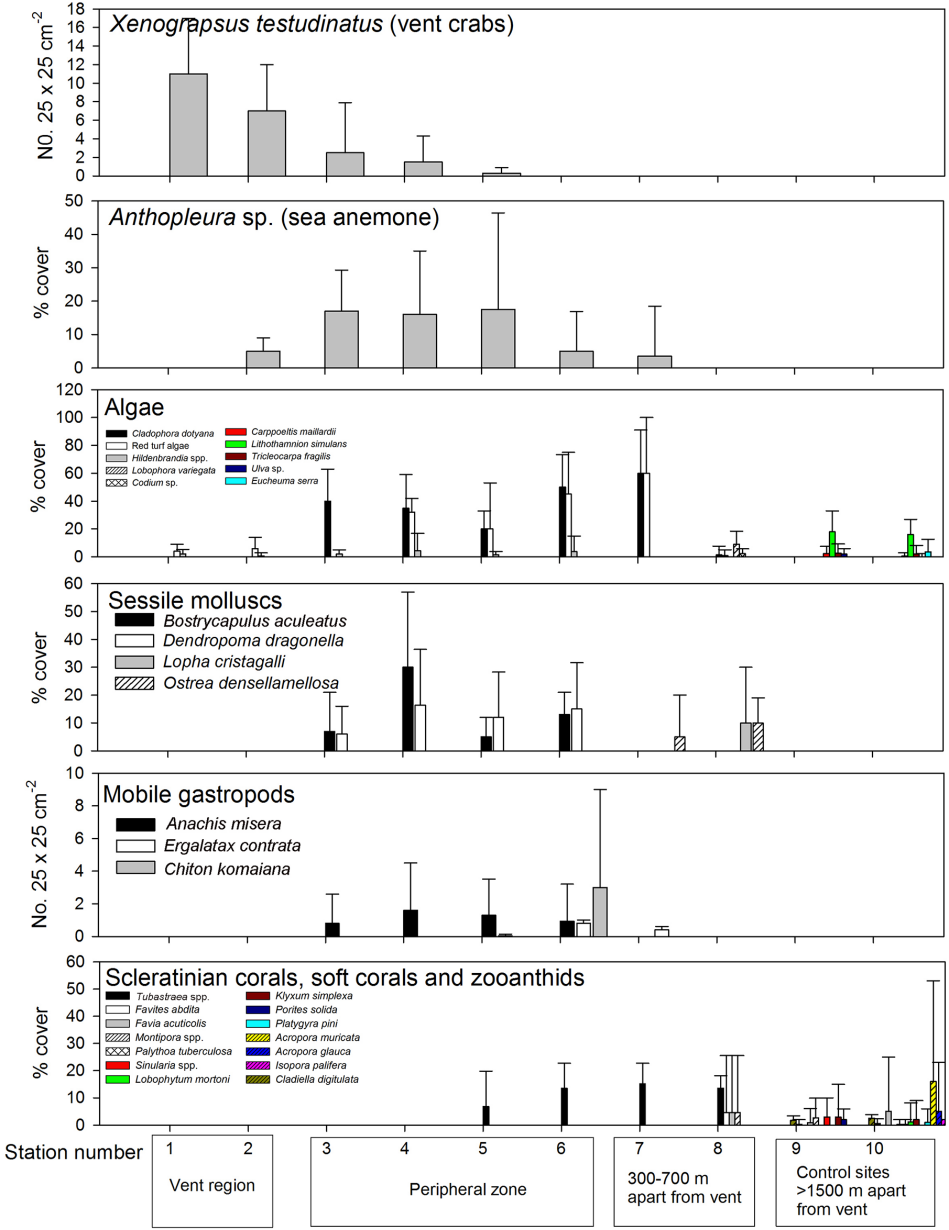
Variation in mean (±1 SD) abundance of major species recorded by transect surveys in Stations 1–8. Species with their maximum mean abundance from all stations < 1 individuals per quadrat or 1% cover were not shown. See S2 Table for a complete list of the species recorded in all stations. Note the red turf algae contains mixture of including *Ceratodictyon repens*, *Chondracanthus intermedius* and *Pterocladilla* sp. which cannot be identify separately in the in-situ quadrat photographs.

**Fig 6:**
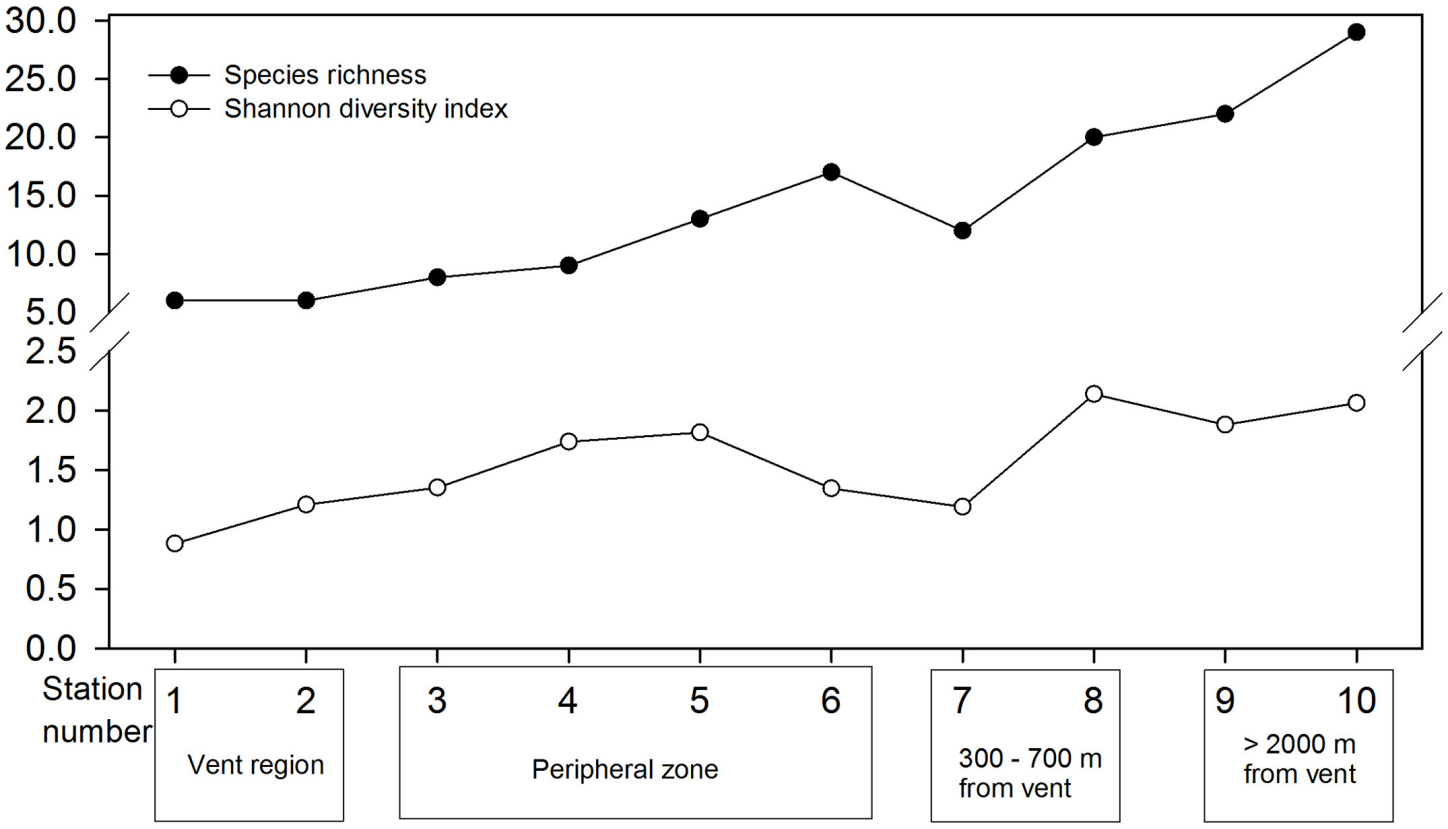
Number of species recorded in each station along the gradient from the vent region (Stations 1–2) to the control stations >2000 metres away (Stations 9 and 10). See S2 Table for a complete list of the species recorded in all stations.

**Fig 7:**
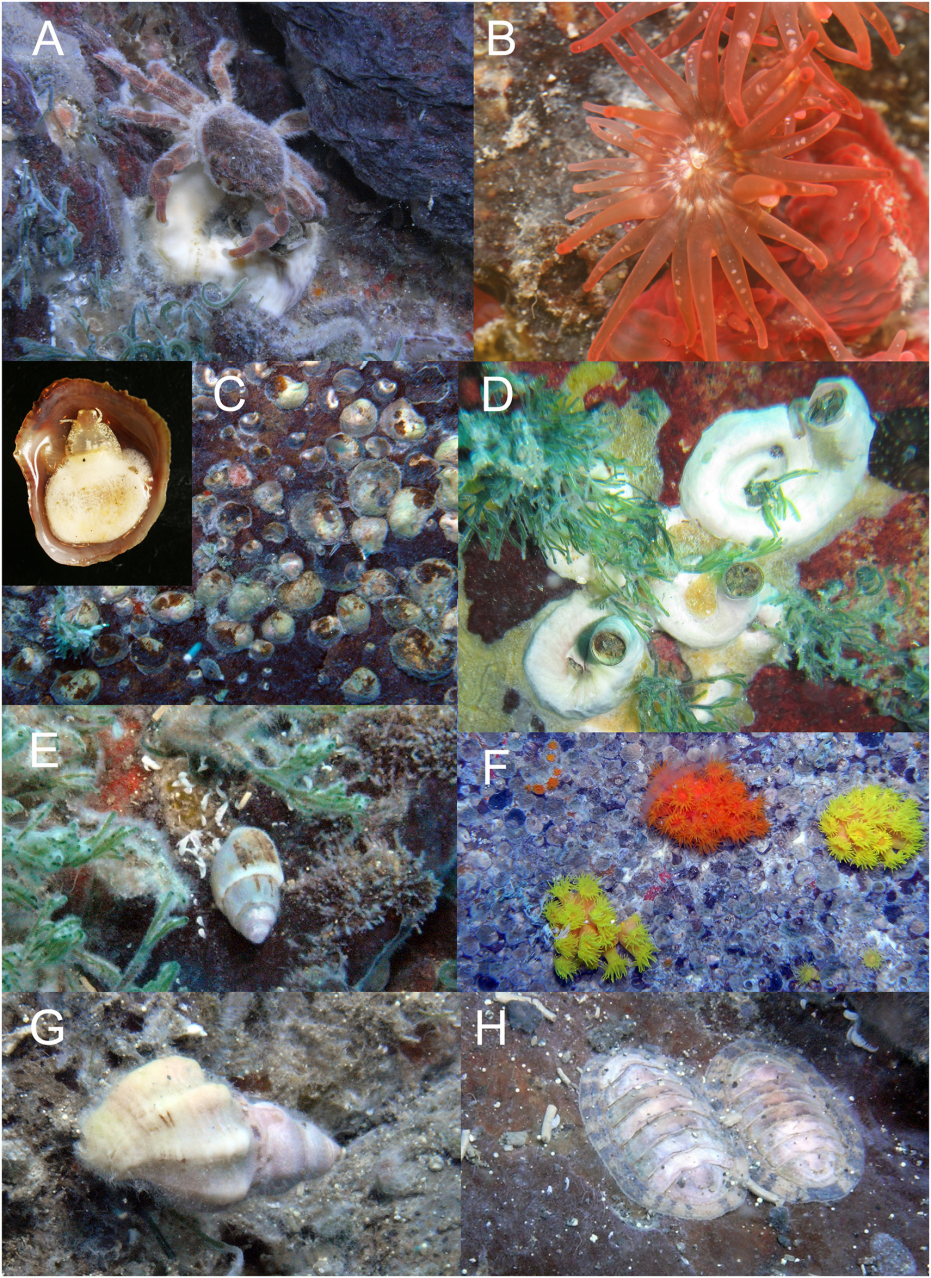
Species found in shallow water vent area off Kueishan Island. (A) Vent crab *Xenograpsus testudinatus* with the carapace covered by bacterial biofilm. (B) The sea anemone *Anthopleura* sp. (C) Sessile gastropod *Bostrycapulus aculeatus*. (D) The mollusk *Dendropoma dragonella* (note the presence of opercular valves distinguished *Dendropoma* from the morphologically similar genus *Serpulorbis*). (E) The carnivorous snail *Anachis misera* associated with the algae *Cladophora dotyana*. (F) The coral *Tubastrea*. (G) The predatory gastropod *Ergalatx contratus*. (H) The chiton, *Chiton komaina*.

The biological assemblages displayed a transition with increasing horizontal distance from the vent region (Figs 5 and 6, S2 Table). The vent crab *Xenograpsus testudinatus* Ng, Huang & Ho, 2000 was the dominant species in the vent region (Stations 1 and 2; Figs 5 and 7A) and appeared in high density (Station 1: 11 ± 6 individuals per 0.0625 m^-2^, Station 2: 7 ± 5 individuals per 0.0625 m^-2^) around the yellow and white vents. Other macrofauna were absence in the major vent stations studied, with the only exception being of the sea anemone *Anthopleura* Duchassaing de Fonbressin & Michelotti, 1860 sp. (Figs 5 and 7B) at Station 2 (5 ± 4% coverage). The rock surfaces of the vent region were mostly bare but occasionally covered with encrusting red algae *Hildenbrandia* Nardo, 1834 spp. and red turf algae including *Ceratodictyon repens* (Kutzing) R.E. Norris, 1987, *Chondracanthus intermedius* (Suringar) Hommersand, 1993, and *Pterocladiella* B. Santelices & Hommersand, 1997 sp. The abundance of vent crab gradually dropped to zero in the peripheral region (Stations 3–6; Fig 5), in which sea anemone *Anthoplerua* sp. became the dominant species and having maximum of 17.5 ± 28.9% coverage in Station 5. (Fig 5). Sessile mollusks, *Bostrycapulus aculeatus* (Gmelin, 1791) (Fig 7C) and *Dendropoma dragonella* (Okutani & Habe, 1975) (Fig 7D), were common in the peripheral region (Fig 5). Numerous other species including carnivorous gastropod *Anachis misera* (Sowerby, 1844) associated with the green algae patches which dominated by *Cladophora dotyana* W. J. Gilbert, 1965 patches (Fig 7E), chiton *Chiton komaiana* Is. & Iw. Taki, 1929 (Figs 5 and 7H), and carnivorous gastropods, *Ergalatax contrata* (Reeve, 1846) (Fig 5) and *Monoplex nicobaricus* (Röding, 1798), were also recorded at Station 6. Furthermore, the coverage of algae increased in the peripheral area, with green algae *Cladophora dotyana* W. J. Gilbert, 1965 (20–50% coverage from stations 3–6) (Figs 5 and 7D) and red turf algae including *Ceratodictyon repens*, *Chondracanthus intermedius*, and *Pterocladiella* sp. (total of 32–60% coverage from stations 3–6) being commonly observed along the substratum.

The species assemblage showed another transition in the area >300 m from the vent (Stations 7 and 8; Fig 5). The sessile mollusk species in the peripheral zone were replaced by oysters, *Lopha cristagalli* (Linnaeus, 1758) (10 ± 20% cover at Station 8) and *Ostrea denselamellosa* Lischke, 1869 (5–10% cover from Stations 7–8). The abundance of green algae *Cladophora dotyana* and red turf algae dramatically decreased over a distance of 700 m from the vent (Station 8; *Cladophora dotyana*: 1.4 ± 6.4%, Red turf: 0.9 ± 4.2%), where low-coverage green algae *Codium* Stackhouse, 1797 sp. (8.3 ± 6%) occurred. Scleractinian coral *Tubastraea* Lesson, 1829 sp. (Fig 7F) were observed first to occur in the outer margin of the peripheral zone (Stations 5 and 6, 6.8–13.6% coverage) and then throughout all of the more distant studied stations (Fig 5). Other reef-building coral species such as *Montipora* Blainville, 1830 spp., *Favia* Milne Edwards, 1857 and zooanthids were observed only 700 m from the vents (Station 8; Fig 5). In the control stations, there are higher diversity of zooanthids and scleractinian coral species and their coverage ranged from 5–20% in Stations 9 and 10.

**Fig 8:**
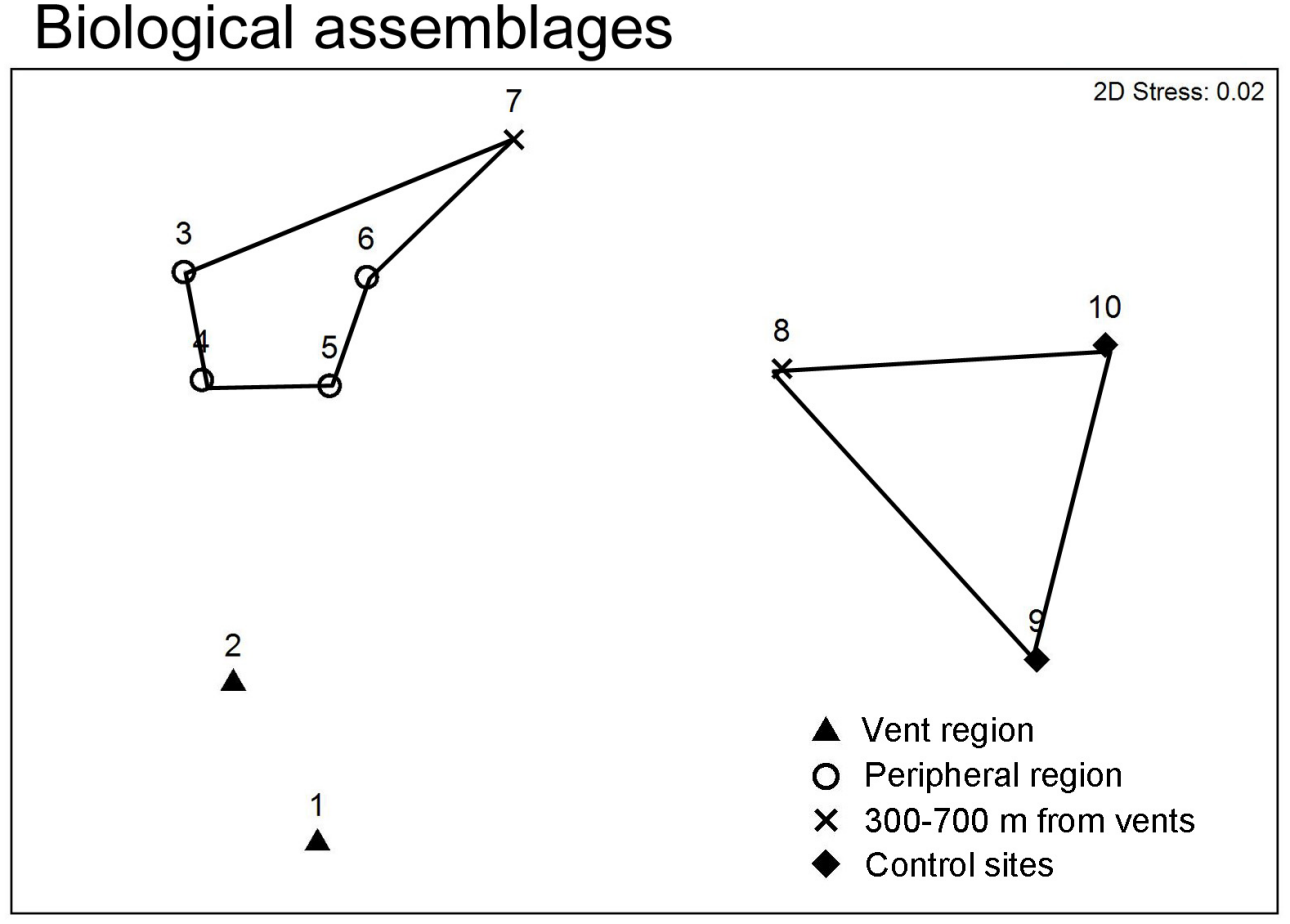
Multivariate nMDS plot of species composition of the Stations 1–8.

Increasing richness of macrofauna and flora diversity was observed with increasing distance from the vent (Fig 6). Less than 10 species were recorded within the vent area based on the transect study, whereas 8–15 species were observed in the peripheral zone. The recorded number of species approached 19 at a distance of 700 m from the vent (Station 8) and reached a maximum of 25 at the studied station most distant from the vent (Station 10: >2000 m from the vent area; Fig 6). Variation in Shannon Index followed similar pattern, with 0.8–1.2 in the vent area (Station 1–2) and increased to 1.8–2.1 in the control stations (Stations 9–10) (Fig 6). Species saturation curve plotted against number of quadrats studied suggested that the trend of increase in number of species recorded have reached a plateau in all stations (S2 Fig). The nMDS plot of the species composition showed that stations were clustered according to their distance from the vent region (Fig 8). Three major clusters were observed: the vent area (Stations 1 and 2), peripheral region (Stations 3–7), Stations 8–10 scattered at farther distance and formed another cluster (Fig 8). The ANOSIM showed significant differences in species compositions among the regions (*R* = 0.98, *p* = 0.005).

The BVSTEP analysis selected the subset of environmental variables: Cl^-^, Mg^2+^, Mn^2+^, SiO_2_, SO_4_^2-^, salinity, temperature pH and mean depth of the station (Table 3), which have the same ordination pattern in the nMDS plot of all environmental variables. From Bio-Env analysis, variation in Ca^2+^, Cl^-^, Temp., pH and depth had highest Spearman correlation coefficient, 0.804, to the changes in the species variations among the 10 stations (Table 3). pH or depth as sole factor can only have 0.3 and 0.4 Spearman’s correlation coefficient to the changes in biological assemblage patterns.

**Table 3:** BIO-ENV results of single and multiple environmental parameters affecting the species distribution pattern.

Environmental parameters	Spearman correlation coefficient
pH	0.321
depth	0.413
pH, depth	0.576
Cl^-^, pH, depth	0.784
Cl^-^, Mg^2+^, Temp., pH, depth	0.785
Ca^2+^, Temp., pH, depth	0.788
Ca^2+^, Cl^-^, Fe^2+, 3+^. Mg^2+^, Temp., pH, depth	0.789
Ca^2+^, Cl^-^, Mg^2+^, Temp., pH, depth	0.794
Ca^2+^, Cl^-^, Temp., pH, depth	0.806

## Discussion

### Physical and chemical properties of the waters off Kueishan Island

In the present study, we documented the physical, chemical, and biological environment of the shallow-water hydrothermal vents off Kueishan Island. The fluid discharged from the vents has a significant impact on water chemistry and hence on the biological composition of the surrounding area. The vent fluid has a higher water temperature and thus is positively buoyant. As a result, fluid discharged from the vents was transported upward and floated on the water surface. The microbubbles of sulfur and carbon dioxide gases as well as aggregates of sulphur bacteria including *Epsilonproteobacteria* and *Gammaproteobacteria* [10], resulting in a whitening of the surface water, whereas the bottom layer remained relatively transparent. Accordingly, the influence of the vent fluid on water chemistries is more pronounced in the surface layer (e.g. with lower pH in surface water when compared to bottom waters; S^2-^ and PO_4_^2-^ was detected in the vent mouths and surface water, but not at bottom waters adjacent to the vent mouths) than in the bottom layer, resulting in vertical differences in the physicochemical properties. The measured surface water chemistry of the vent region was more similar to the fluid from the smokers than water of the bottom layer, even as close as 1 m to the smokers near the vents at the bottom. Chen et al. [8, 9] showed that the temperature of seawater around the vent are correlated with diurnal tidal patterns, further suggesting the vent fluids are rapidly circulated by surface currents. Accordingly, we expect a stronger negative impact on species in the surface water than in the bottom layer, which is consistent with observations that plankton in the surface water are killed by vent plumes and produce “marine snow” comprised of the falling dead plankton bodies [26]. Furthermore, experimental evidence from planktonic copepods caged at different depths (1, 6, and 13 m) in the Kueishan Island vent region showed that the copepods at the top layer had the greatest mortality [27]. By contrast, the benthos is less influenced by the hydrothermal vent fluid and supports a low richness of calcareous fauna (e.g., gastropods and crabs).

At the horizontal scale, we determined a reduced pH values and dissolved oxygen levels closer to the vent area. By contrast, the vent emission exerted no significant effect on water temperature (except within 1–2 m of the smokers, temperature can reach 32–35°C) or salinity. The concentrations of measured inorganic compound and metal ions were highly variable across the stations and didn’t significantly differ, except for arsenic (which has a higher concentration near the vent stations) and manganese ion (which occurred only in vent water).

The water chemistries measured in the present study reflect the environmental conditions only at one particular time interval. A previous study reported that high temporal variations in water temperature off Kueishan Island are attributable to diurnal tides [9]; therefore, it is reasonable to anticipate a similar level of variation in other parameters. The complex surface circulation may transport different amounts of vent fluid to individual stations in the same region (e.g., peripheral region), resulting in the dynamic pattern observed among the stations. Nevertheless, although the temporal variations require further investigation, our results showed clear differences in the water chemistries of the major vent area, its peripheral region, and the stations farther away.

### Biological assemblages

Species richness was positively correlated with distance from the vent, in agreement with other studies on shallow-water vent systems [28–34]. It is unsurprising that the influence of the vent decreases with increasing horizontal distance because of the dilution and lower concentration of toxicants by sea water. However, previous studies on shallow-water vents have examined generally short spatial scales of a few meters to a few hundred meters around the vents [29, 33, 34] and consequently have been unable to fully reflect the range influenced by vent fluid. In the present study, we surveyed a horizontal distance extending approximately 2000 m from the vent and so effectively show the spatial pattern of the shallow-water vent assemblages off Kueishan Island. We determined that the upward trend in species richness continued throughout all studied stations and that a greater number of species were observed in locations 1500–2000 m from the vents, indicating that vent fluids may exert a negative impact over more than 1–2 km. This large scale of influence was unexpected because the vent fluid was assumed to be quickly diluted by the large amount of sea water. Therefore, a larger spatial setting may be required in future studies on shallow-water vent ecologies.

The community structure off Kueishan Island displayed numerous transitions with increasing horizontal distance from the vents. These transitions were supported by a cluster pattern representing the biological compositions of the stations on the nMDS plot, which showed three major groups (i.e., vent region, the peripheral zone, and stations >700 m from the vent). These clusters were broadly congruent with changes in environmental conditions, despite greater discrepancies observed among stations on an nMDS plot of environmental factors. The vent region was dominated by vent crabs *X*. *testudinatus*, which is an omnivorous species [26, 35, 36]. *Xenograpsus testudinatus* is physically adapted to the vent environment and possesses proteolytic enzymes (an adaption for irregular food availability) that are active over wide ranges of temperature and pH values, even in the presence of heavy-metal inhibitors [37]. *Xenograpsus testudinatus* can also store large amounts of lipids for survival in periods of food scarcity [37]. The low diversity of algae in the vent region, which was inhabited only by patches of brown encrusting algae *Ralfsia* sp., can likely be attributed to the low pH level [38, 39]. Calcified algae is absent in the vent water and were only found in the control stations only (e.g. *Lithothamnion simulans*). This observation is consistent with previous study on shallow water vent showed that reduced pH near the vent will impede the growth and survival of calcified algae [31]. Furthermore, chromophoric dissolved oxygen (CDOM) from white vents absorbs sunlight in the same quanta as aquatic plants, including phytoplankton and algae, and hence impedes photosynthesis and algal growth [40, 41].

Suspension feeders including the sea anemone *Anthopleura* sp., slipper shell *Bostrycapulus aculeatus*, and worm shell *Dendropoma dragonella* were major inhabitants of regions peripheral to vents [35, 42]. The studied vent peripheral region supported a high coverage of the green algae *Cladophora dotyana* and red turf algae. Therefore, some grazers, though lower in abundance than the suspension feeders, were also observed. For instance, the cowrie *Cypreae* Linnaeus 1758 spp., chiton *Chiton komaiana*, and omnivorous gastropod *Anachis*, which feed on dead animals in addition to algae, were observed. A previous study showed that vent fluid off Kueishan Island promoted erosion in the vertical rods and radial bars of the scleractinian coral *Acropora valida*, suggesting that vent water acidity is a major factor limiting coral survival [43].

Almost all of the species observed within the major vent region and peripheral zone were replaced by other species at distances more than 300 m from the vents. Scleractinian coral *Tubastraea* sp. occurred first in the outer margin of the peripheral region and were present in high abundance 300–700 m from the vent region. *Tubastraea* corals have no zooxanthellae for photosynthesis in their tissue and are suspension feeders in well-circulated water [44]. *Tubastraea* is a rapidly growing species [45] that can quickly colonize vacant spaces. Acidic water and the blocking of sunlight by CDOM appear to be prohibiting the survival of other coral species. Therefore, *Tubastraea*, which generally live in shaded areas, become the dominant coral species on an open rock surface in the absence of competition. Fish starting to be observed from stations 8–10 during the transect studies which include *Heniochus acuminatus* (Linnaeus, 1758), *Chaetodon vagabundus* Linnaeus, 1758, *Chaetodon auripes* Jordan & Snyder, 1901, *Lutjanus kasimira* (Forsskal, 1775), *Thalassoma lunare* (Linnaeus, 1758) and *Diodon holocanthus* Linnaeus, 1758.

Among the physical and chemical parameters measured in the present attempt, variation in Ca^2+^, Cl^-^, temperature, pH and depth were revealed to show the strongest correlation with the change in benthic community structure, suggesting multiple factors of vent fluid were influencing the associated fauna. In a study on the effect of acidification by volcanic CO_2_ from shallow water, Hall-Spencer et al. [31] found that typical rocky shore communities with abundant calcareous organisms at normal pH water (8.1–8.2) shifted to communities lacking scleractinian corals with significant reductions in sea urchin and coralline algal abundance at lower pH water near the vent (mean 7.8–7.9, minimum 7.4–7.5). The benthic community in Kueishan Island show similar transition under the impact of lower pH (and possibly other carbonate compounds that were not analyzed in the present attempt) along the horizontal gradient of the vent. Only very limited number of species can survive around the vent mouth (the vent crab) and subsequently non-calcifying autotrophs (the sea anemone) increase in abundance in more peripheral water of which the toxic effects of the vents diminish. Non-calcified algae thrive in the peripheral region in the absence of completion and grazing from calcifiers (clarified algae and gastropods) in low pH water. The algae coverage decrease when move further away from the vent when calcareous gastropods and calcifying algae capable to survive. The coverage of scleractinia coral also increase. Therefore, it appears that the transitions of benthic community is a result of tolerance to toxic vent water and biological interactions. In the present study, there are no clear trends in the spatial variation of physico-chemical parameters departing from the vent region, probably due to limited temporal samplings. It is, therefore, difficult to correlate the spatial variation of physico-chemical parameters to the change in biological communities. Further studies should involve intensive temporal and spatial samplings of water waters and its relations to the community in the Kueishan Island shallow water vent system.

The macrofauna off Kueishan Island is mainly composed of suspension feeders and some scavengers. “Marine snow,” which comprises plankton killed by plumes from the vents (suspended mainly by the regional currents in the station), may not only provide a major food source to the vent crab *X*. *testudinatus* [26] but also support a high density of other suspension feeders. Similarly, scavengers, such as *Ergalatax* and *Anachis*, may feed on the dead plankton and other larger organisms killed by the vent fluids. The green algae *Cladophora dotyana* and red turf algae, although in extremely low abundance, were observed even in proximity to the smokers. The algae occurred in high abundance in the peripheral region (>40% coverage), suggesting that photosynthetic reactions contribute a considerable proportion of the primary production in this shallow-water vent system. However, the abundance of grazers (*Cypraea* and *Chiton*) was unexpectedly low. Therefore, the algal population in the vent area appears not to be strongly controlled by grazing but limited by environmental factors including reduced solar radiation because of the whitish water surface and CDOM in the water. Although Tang et al. [10] reported several chemosynthetic bacteria in the vent fluid and surface waters, we could not determine the link between these bacteria and a higher trophic group because none of the recorded macrofauna displays an apparent dependence on the bacteria as a food source. Future research on macrofauna may determine the energy transfer link between chemosynthetic bacteria and macrofauna.

Shallow-water vents are typically inhabited by a subset of opportunistic taxa from proximal habitats, instead of the vent obligate specialists that inhabit deep-sea hydrothermal vents [32, 34]. A similar phenomenon was observed off Kueishan Island in that all but one invertebrate species inhabiting the vent and peripheral zone were common shallow-water species in non-vent habitats. The only exception was from *X*. *testudinatus*. *Xenograpsus testudinatus* was also recorded in deep-sea vents of Taiwan [46] and shallow-water vents of Japan [47]. Although the preferred habitat of the species in deeper water remains uncertain, its cogeneric, *X*. *novaeinsularis* Takeda & Kurata, 1977, was also reported in vent habitats [48]. Therefore, the genus is likely vent obligate fauna. Further study of meiofauna and zooplankton in waters off the island may show more vent obligate fauna.

The present study shows how sub-tidal community changes along an acidity gradient. Our results provide an *in-situ* example to demonstrate how sub-tidal community response to the effect of ocean acidification. Abundance of corals will decrease whilst the proportion of encrusting brown and green algae will increase under the scenario of ocean acidification. Hall-Spencer et al. [31] investigated the change in rocky intertidal community along the pH gradient from a shallow water cold vent in Ischia, Italy. Hall-Spencer et al. [31] showed that calcareous organisms including coralline algae and sea urchins decreased in abundance in the lower pH regions. In Ischia, gastropods living close to the vents regions suffered from severe erosion in shells, resulted in eroded and pitted shells [31]. Similar observations were also reported in the snail *Anachis* in the Kueishan Island. Chen et al. [12] showed that the snail *Anachis* has more globular shells in the vent regions than at the surrounding region, indicating lower pH waters will negatively impact the shell growth in molluscs. Further study should investigate how the gradient of total alkalinity and Dissolved Inorganic Carbon (DIC) affect community patterns in order to achieve a more accurate prediction of future community responses to the effect of ocean acidifiation.

## Supporting Information

S1 Table Test kit product number, pH working range and sensitivity range of the ions tested in the present study. (DOCX)

S2 Table List of species recorded from transect surveys in the ten stations (+ = presences, − = absences). (DOCX)

S1 Fig Saturation curve of number of species recorded against number of transect surveyed. (TIF)

S2 Fig Variations in mean (+1SD) chemical parameters at the mouth of the yellow (YV) and white (WV) vents, their adjacent waters at 1 m and the surface water above vents. (TIF)

S1 Text Physicochemical environment of the water at the vent mouth, surrounding water and surface water. (DOCX)
