# Supplementary material for: Community Structure of Macrobiota and Environmental Parameters in Shallow Water Hydrothermal Vents off Kueishan Island, Taiwan
Source: PLoS One. 2016 Feb 5;11(2):e0148675. doi: 10.1371/journal.pone.0148675 (PMC4744018; doi:10.1371/journal.pone.0148675)
Supplement: S1 Table — (DOCX) [file pone.0148675.s001.docx]

S1 Table. Test kit product number, pH working range and sensitivity range of the ions tested in the present study.

|  | Test Kit no. | pH range | Sensitivity range |
| --- | --- | --- | --- |
| Aluminum test ( Al^3+^) | 114825 | 3-10 | 0.02-0.2mg/l |
| Calcium test ( Ca^2+^) | 114815 | 4-10 | 10-160mg/l |
| Chloride test ( Cl^-^) | 114897 | 1-12 | 10-250mg/l |
| Iron test (Fe(II)+(III) | 100796 | 2-8 | 0.01-1mg/l |
| Magnesium cell test ( Mg^2+^) | 100815 | 3-9 | 5.0-75.0mg/l |
| Manganese test (Mn^2+^) | 114770 | 2-7 | 0.01-2mg/l |
| Nitrate test (NO_3_^-^) | 114942 | 1-3 | 0.9-75.3mg/l |
| Phosphate test (PO_4_^3-^) | 114848 | 0-10 | 0.03-3.07mg/l |
| Sulfide test ( S^2-^) | 114779 | 2-10 | 0.1-1.5 mg/l |
| Silicate test ( SiO_2_) | 114794 | 2-10 | 0.21-10.7 mg/l |
| Sulfate cell test ( SO_4_^2-^) | 114548 | 2-10 | 5-250 mg/l |
