# Supplementary material for: Community Structure of Macrobiota and Environmental Parameters in Shallow Water Hydrothermal Vents off Kueishan Island, Taiwan
Source: PLoS One. 2016 Feb 5;11(2):e0148675. doi: 10.1371/journal.pone.0148675 (PMC4744018; doi:10.1371/journal.pone.0148675)
Supplement: S2 Table — (DOCX) [file pone.0148675.s002.docx]

S4 Table. List of species recorded from transect surveys in the ten stations (+ = presences, - = absences).

|  | Common name | Stn. 1 | Stn. 2 | Stn. 3 | Stn.4 | Stn. 5 | Stn. 6 | Stn. 7 | Stn.8 | Stn. 9 | Stn. 10 |
| --- | --- | --- | --- | --- | --- | --- | --- | --- | --- | --- | --- |
|  |  |  |  |  |  |  |  |  |  |  |  |
| **Crustacea** |  |  |  |  |  |  |  |  |  |  |  |
| *Xenograpsus testudinatus* Ng, Huang & Ho, 2000 | vent crab | + | + | + | + | + | - | - | - | - | - |
| *Macromedaeus distinguendus* (De Haan, 1835) | Crab | - | - | - | + | + | + | - | - | - | - |
| *Phymodius* sp. Milne-Edwards, 1863 | crab | - | - | - | - | - | - | - | + | - | - |
| *Dardanus pedunculatus* (Herbst, 1804) | Hermit crab | - | - | - | - | - | - | - | + | - | - |
| *Calcinus vachoni* Forest, 1958 | Hermit crab | - | - | - | - | - | - | - | + | - | - |
| *Alpheus edwardsii* (Audouin, 1826) | Piston shrimp | - | + | - | - | - | - | - | + | - | - |
| *Percnon planissimum* (Herbst, 1804) | crab | - | - | - | - | - | - | - | + | - | - |
|  |  |  |  |  |  |  |  |  |  |  |  |
| **Mollusca** |  |  |  |  |  |  |  |  |  |  |  |
| *Dendropoma dragonella* (Okutani & Habe, 1975) | Snake snail | - | - | + | + | + | + | - | - | - | - |
| *Ceraesignum maximum* (Sowerby, 1825) | Snake snail | - | - | - | - | - | - | - | - | + | - |
| *Bostrycapulus aculeatus* (Gmelin, 1791) |  | - | - | + | + | + | + | - | - | - | - |
| *Anachis miser* (Sowerby, 1844) | snail | - | - | + | + | + | + | - | - | - | - |
| *Monetaria annulus* (Linnaeus, 1758) | Cowrie | - | - | - | - | - | + | - | - | + | - |
| *Erronea onyx* (Linnaeus, 1758) | Cowrie | - | - | - | - | - | - | - | - | + | - |
| *Ergalatax contracta* (Reeve, 1846) | snail | - | - | - | - | - | + | + | - | - | - |
| *Pyrene punctata* (Bruguière, 1789) | snail | - | - | - | - | - | + | - | - | - | - |
| *Pardalinops testudinaria* (Link, 1807) | snail | - | - | - | - | - | - | + | - | + | - |
| *Chiton komaianus* Is. & Iw. Taki, 1929 | chiton | - | - | - | - | - | + | - | - | - | - |
| *Monoplex nicobaricus* (Röding, 1798) | snail | - | - | - | - | - | + | + | - | - | - |
| *Ostrea denselamellosa* Lischke, 1869 | Oyster | - | - | - | - | - | - | + | + | - | - |
| *Lopha cristagalli* (Linnaeus, 1758) | Oyster | - | - | - | - | - | - | - | + | - | - |
|  |  |  |  |  |  |  |  |  |  |  |  |
| **Polychaeta** |  |  |  |  |  |  |  |  |  |  |  |
| *Nereinid* spp. Levinsen, 1883 | polychaetes | + | + | - | - | - | - | + | + | + | - |
|  |  |  |  |  |  |  |  |  |  |  |  |
| **Echinoderms** |  |  | - |  |  |  |  |  |  |  |  |
| *Echinaster luzonicus* (Gray, 1840) | sea star | - | - | - | - | - | - | - | - | + | + |
|  |  |  |  |  |  |  |  |  |  |  |  |
| **Cnidaria** |  |  |  |  |  |  |  |  |  |  |  |
| Actiniaria (sea anenones) |  |  |  |  |  |  |  |  |  |  |  |
| *Anthopleura* sp. Duchassaing de Fonbressin & Michelotti, 1860 | sea anemone | - | - | + | + | + | + | + | - | - | - |
| *Anthopleura* sp. 2 Duchassaing de Fonbressin & Michelotti, 1860 | sea anemone | - | - | - | - | - | - | - | - | + | - |
| *Discosoma* sp. Rüppell & Leuckart, 1828 |  |  |  |  |  |  |  |  |  |  |  |
| *Zoantharia (Zooanthes)* |  |  |  |  |  |  |  |  |  |  |  |
| ?*Palythoa yongei* Carlgren, 1937 |  | - | - | - | - | - | - | - | - | + | - |
| *Palythoa tuberculosa* (Esper, 1791) |  | - | - | - | - | - | - | - | + | + | + |
|  |  |  |  |  |  |  |  |  |  |  |  |
| Hydrocorallia (fire corals) |  |  |  |  |  |  |  |  |  |  |  |
| *Millepora exaesa* Forsskål, 1775 |  | - | - | - | - | - | - | - | - | + | - |
|  |  |  |  |  |  |  |  |  |  |  |  |
| **Corals** |  |  |  |  |  |  |  |  |  |  |  |
| *Tubastraea coccinea* Lesson, 1829 |  | - | - | - | - | + | + | + | + | + | + |
| *Tubastraea micranthus* (Ehrenberg, 1834) |  | - | - | - | - | + | + | + | + | + | - |
| *Favites abdita* (Ellis & Solander, 1786) |  | - | - | - | - | - | - | - | + | - | - |
| *Favites acuticollis* (Ortmann, 1889) |  | - | - | - | - | - | - | - | + | - | - |
| *Montipora turgescens* Bernard, 1897 |  | - | - | - | - | - | - | - | + | + | - |
| *Porites solida* (Forskål, 1775) |  | - | - | - | - | - | - | - | - | - | + |
| *Acropora glauca* (Brook, 1893) |  | - | - | - | - | - | - | - | - | - | + |
| *Dipsastraea favus (*Forskål, 1775) |  | - | - | - | - | - | - | - | - | + | + |
| *Seriatopora hystrix* Dana, 1846 |  | - | - | - | - | - | - | - | - | - | + |
| *Platygyra pini* Chevalier, 1975 |  | - | - | - | - | - | - | - | - | + | + |
| *Isopora palifera* (Lamarck, 1816) |  | - | - | - | - | - | - | - | - | + | + |
| *Acropora muricata* (Linnaeus, 1758) |  | - | - | - | - | - | - | - | - | - | + |
|  |  |  |  |  |  |  |  |  |  |  |  |
| **Soft corals** |  |  |  |  |  |  |  |  |  |  |  |
| *Cladiella digitulatum* (Klunzinger, 1877) |  | - | - | - | - | - | - | - | - | + | - |
| *Lobophytum mortoni* Benayahu & van Ofwegen, 2009 |  | - | - | - | - | - | - | - | - | - | + |
| *Sinularia brassica* May, 1898 |  | - | - | - | - | - | - | - | - | - | + |
| *Sinularia exilis* Tixier-Durivault, 1970 |  | - | - | - | - | - | - | - | - | - | + |
| *Sinularia* sp. May, 1898 |  | - | - | - | - | - | - | - | - | - | + |
| *Klyxum simplex* (Thomson & Dean, 1931) |  | - | - | - | - | - | - | - | - | - | + |
|  |  |  |  |  |  |  |  |  |  |  |  |
| **Sponges** |  |  |  |  |  |  |  |  |  |  |  |
| *Haliclona (Haliclona)* sp. Grant, 1836 |  | - | - | - | - | - | - | - | - | + | *-* |
|  |  |  |  |  |  |  |  |  |  |  |  |
| **Algae** |  |  | - |  |  |  |  |  |  |  |  |
| *Hildenbrandia* spp. |  | + | + | + | + | + | + | + | + | - | - |
| Red turf |  | + | + | + | + | + | + | - | - | - | - |
| *Gelidiella* sp. Feldmann & G.Hamel, 1934 |  | + | + | + | + | + | + | + | + | - | + |
| *Carpopeltis maillardii* (Montagne & Millardet) Chiang, 1970 |  | - | - | - | - | - | - | - | + | - | + |
| *Bornetella sphaerica* (Zanardini) Solms-Laubach, 1892 |  | - | - | - | - | - | - | - | - | - | + |
| *Exallosorus harveyanus* (Pappe ex Kützing) Phillips, 1997 |  | - | - | - | - | - | - | - | - | - | + |
| *Lobophora variegata* (Lamouroux) Womersley ex Oliveira, 1977 |  | - | - | - | - | - | - | - | - | - | + |
| *Cladophora dotyana* Gilbert, 1965 |  | + | + | + | + | + | + | + | + | - | + |
| *Titanophycus validus* (Harvey) Huisman, Saunders & Sherwood, 2006 |  | - | - | - | - | - | - | - | - | - | + |
| *Eucheuma* sp. Agardh, 1847 |  | - | - | - | - | - | - | - | - | - | + |
| *Tricleocarpa fragilis* (Linnaeus) Huisman & Townsend, 1993 |  | - | - | - | - | - | - | - | - | + | + |
| *Mesophyllum simulans* (Foslie) Me.Lemoine, 1928 |  | - | - | - | - | - | - | - | - | + | + |
| *Codium* sp. Stackhouse, 1797 |  | - | - | - | - | - | - | - | - | - | + |
| *Ulva* sp. Linnaeus, 1753 |  | - | - | - | - | - | - | - | - | + | + |
| *Dictyopteris repens* (Okamura) Børgesen, 1924 |  | - | - | - | - | - | - | - | - | - | + |
| *Gracilaria* sp. Greville, 1830 |  | - | - | - | - | - | - | - | - | + | + |
|  |  |  |  |  |  |  |  |  |  |  |  |
| \| Red turf including a mixture of *Ceratodictyon repens* (Kützing) R.E.Norris, 1987, *Chondracanthus intermedius* (Suringar) Hommersand, 1993, and *Pterocladilla* \| \| --- \|   sp. which cannot be quantified in quadrat photos. | | | | | | | | | | | |
