# Supplementary figures and images for: Community Structure of Macrobiota and Environmental Parameters in Shallow Water Hydrothermal Vents off Kueishan Island, Taiwan
Source: PLoS One. 2016 Feb 5;11(2):e0148675. doi: 10.1371/journal.pone.0148675 (PMC4744018; doi:10.1371/journal.pone.0148675)

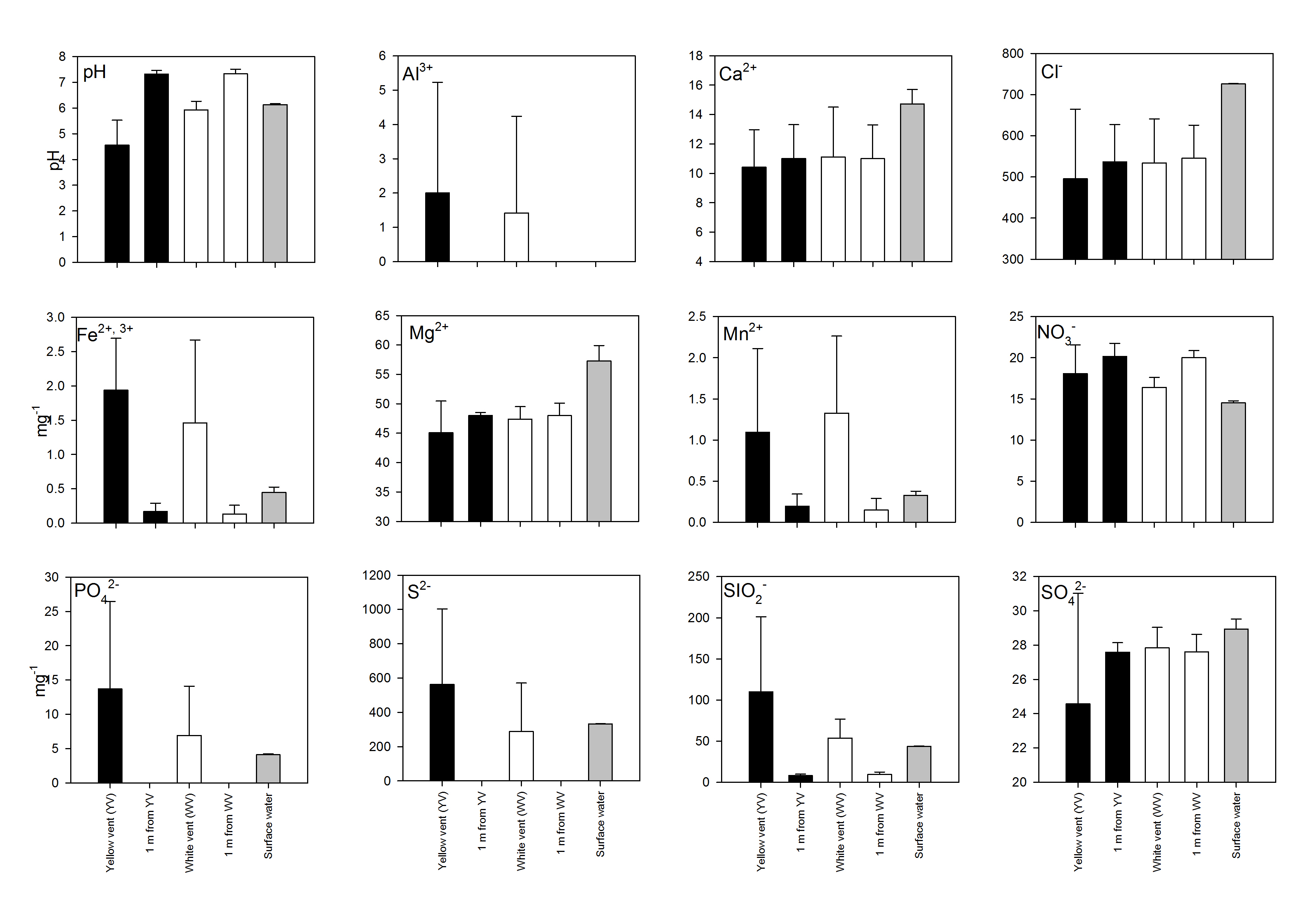

Supplement: S1 Fig — (TIF) [file pone.0148675.s003.tif]

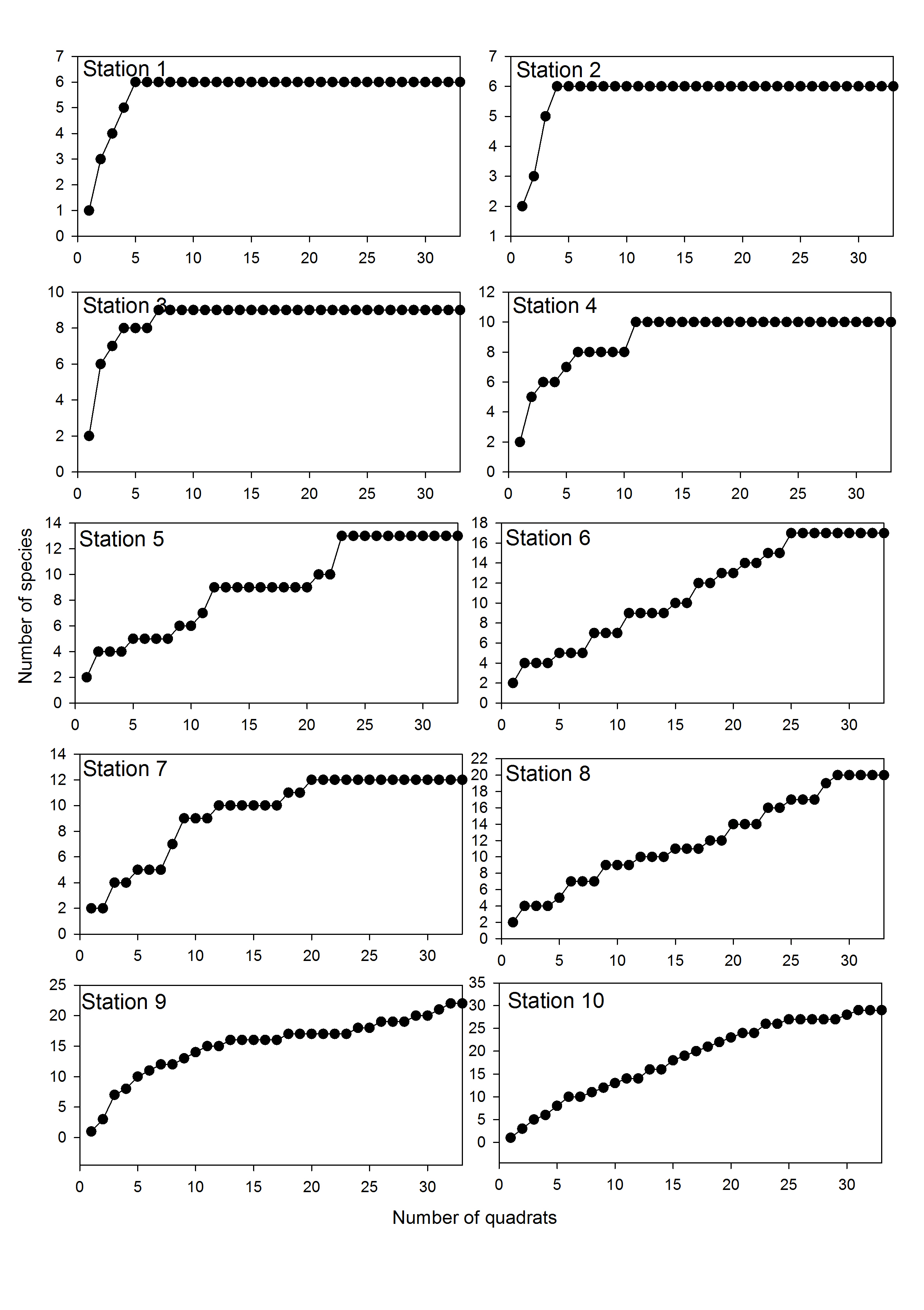

Supplement: S2 Fig — (TIF) [file pone.0148675.s004.tif]
