# Supplementary material for: Community Structure of Macrobiota and Environmental Parameters in Shallow Water Hydrothermal Vents off Kueishan Island, Taiwan
Source: PLoS One. 2016 Feb 5;11(2):e0148675. doi: 10.1371/journal.pone.0148675 (PMC4744018; doi:10.1371/journal.pone.0148675)
Supplement: S1 Text — (DOCX) [file pone.0148675.s005.docx]

**Supplementary materials:**

*Physicochemical environment of the water at the vent mouth, surrounding water and surface water*

From one-way ANOVA, concentration of Ca^2+^ (F (4, 10) = 12.6, p < 0.05) Cl^-^ (F (4, 10) = 283.5, p < 0.05), Fe^2+, 3+^ ((F (32.2, 10) = 152.5, p < 0.05) Mg^2+^ (F (4, 10) = 20.3, p < 0.05) Mn^2+^ (F (4, 10) = 152.5, p < 0.05) NO_3_^-^ ((F (4, 10) = 43.2, p < 0.05) and SiO_2_ (F (4, 10) = 8.3, p < 0.05) was significantly different among the fluids from the yellow and white vents, 1 m apart from both vents and on the surface water. Mn^2+^ and SO_4_^2-^ did not differ among regions. Among the cations and anions tested, yellow vents have significantly greater concentration of Fe^2+, 3+^ (yellow vent: 1.93 ± 0.75 , white vent: 1.46 ± 1.2 mmol kg^-1^) and SiO_2_ (yellow vent: 110.0 ± 91.0 , white vent: 53.8 ± 22.9 mmol kg^-1^) than white vents (p < 0.05, SNK tests), whilst concentrations of others ions were similar between the two vents (Supplementary Fig. 1).

Comparing the physicochemical environment of the vent mouths and waters at 1 m apart from the vents, Al^3+^, PO4^2-^, and S^2-^ were only present in fluids from the yellow and white vents but absent from the waters at 1 m apart from the yellow and white vents. From SNK tests, fluids from the yellow vents contained significantly higher concentration of Fe^2+, 3+^ (yellow vents: 1.93 ± 0.75, 1 m apart from yellow vents: 0.1 ± 0.12 mmol kg^-1^) and SiO_2_ (yellow vents: 110.0 ± 91, 1 m apart from yellow vents: 8.2 ± 1.8 mmol kg^-1^) but lower concentration of NO_3_^-^ (yellow vents: 18.0 ± 3.4 m apart from yellow vents: 20.1 ± 1.6 mmol kg^-1^) from the waters at 1 m apart from the yellow vent. Fluids from the mouth of the white vents have significantly greater concentration of SiO_2_ (white vents: 53.8 ± 22.9 m apart from white vents: 9.3 ± 2.9 mmol kg^-1^) but lower concentration of NO_3_^-^ (white vents: 16.3 ± 1.2 m apart from white vents: 20.0 ± 0.8 mmol kg^-1^) from the waters at 1 m apart from the yellow vent (Supplementary Fig. 1).

From SNK pairwise comparisons, the surface water above the vent region have significantly higher concentration of Ca^2+^, Mg^2+^, Cl-, and lower concentration of NO_3_^-^ than the fluid collected from the white and yellow vents and waters at 1 m apart from yellow and white vents. Arsenic in the fluids from the mouths of yellow (0.03 mgl^-1^) appears to be higher than the fluid collected at the mouth of the white vents (0.002 mgl^-1^) and the waters at 1 m apart from the yellow and white vents (0.002 mgl^-1^ for both regions) (Supplementary Fig. 1).
